# A clinically applicable and generalizable deep learning model for anterior mediastinal tumors in CT images across multiple institutions

**DOI:** 10.1038/s41598-026-37504-z

**Published:** 2026-01-30

**Authors:** Chihiro Takemura, Mototaka Miyake, Kazuma Kobayashi, Hiromi Matsumoto, Ryota Shibaki, Atsushi Urikura, Yasushi Goto, Yasushi Yatabe, Shun-ichi Watanabe, Miyuki Sone, Masahiko Kusumoto, Ryuji Hamamoto, Hirokazu Watanabe

**Affiliations:** 1 Department of Thoracic Surgery, National Cancer Center Hospital, 5-1-1 Tsukiji, Chuo-ku, 104-0045 Tokyo Japan; 2 Department of Diagnostic Radiology, National Cancer Center Hospital, 5-1-1 Tsukiji, Chuo-ku, 104-0045 Tokyo Japan; 3 Division of Medical AI Research and Development, National Cancer Center Research Institute, 5-1-1 Tsukiji, Chuo-ku, 104-0045 Tokyo Japan; 4 Digital Content and Media Sciences Research Division, National Institute of Informatics, 2-1-2 Hitotsubashi, Chiyoda-ku, 101-8430 Tokyo Japan; 5 AI Medical Engineering Team, RIKEN Center for Advanced Intelligence Project, 1-4-1 Nihonbashi, Chuo-ku, 103-0027 Tokyo Japan; 6 Division of Radiological Sciences, Graduate School of Health Sciences, Ibaraki Prefectural University of Health Sciences, 4669-2, Ami-machi, Inashiki-gun, 300-0394 Ibaraki Japan; 7 Department of Thoracic Oncology, National Cancer Center Hospital, 5-1-1 Tsukiji, Chuo-ku, 104-0045 Tokyo Japan; 8 Department of Diagnostic Pathology, National Cancer Center Hospital, 5-1-1 Tsukiji, Chuo-ku, 104-0045 Tokyo Japan

**Keywords:** Anterior mediastinal tumors, Deep learning, No-code AI platform, Segmentation, Detection, Cancer imaging, Cancer imaging, Information technology

## Abstract

Rare diseases are often difficult to diagnose, and their scarcity also makes it challenging to develop deep learning models for them due to limited large-scale datasets. Anterior mediastinal tumors—including thymoma and thymic carcinoma—represent such rare entities. A few diagnostic support systems for these tumors have been proposed; however, no prior studies have tested them across multiple institutions, and clinically applicable and generalizable models remain lacking. A total of 711 computed tomography (CT) images were collected from 136 hospitals, each from a different patient with pathologically proven anterior mediastinal tumors (339 males, 372 females). Of these, 485 images were used for training, 62 for tuning, and 164 for external testing. The external testing dataset comprised CT images from 121 unique institutions not involved in the other datasets. A 3D U-Net-based model was trained on the training dataset, and the model with the best performance on the tuning dataset was selected. This model was then evaluated on the external testing dataset for its segmentation and detection performance across different institutions. Based on the reference standards provided by board-certified diagnostic radiologists, the trained model achieved average Dice scores of 0.82, Intersection over Union (IoU) of 0.72, Precision of 0.85, and Recall of 0.82 for tumor segmentation at the CT-image level. The free-response receiver operating characteristic curve—derived from lesion-wise IoU thresholds—demonstrated high sensitivity and a low false-positive rate for tumor detection. Even under a stricter IoU threshold of 0.50, the model maintained a sensitivity of 0.87 with only 0.61 false positives per scan. Our model achieved clinically applicable segmentation and detection performance for anterior mediastinal tumors, demonstrating broad generalizability across 121 institutions and overcoming the data-scarcity challenges inherent to such rare diseases.

## Introduction

Anterior mediastinal tumors, such as thymoma and thymic carcinoma, are rare entities that pose substantial diagnostic challenges due to their low incidence and distinctive radiological features^1–3^. While computed tomography (CT) remains the cornerstone for imaging evaluation, precise interpretation often demands substantial expertise and experience, even though accurate pretreatment diagnosis is essential for effective treatment planning and prognostic assessment^4^. Hence, there is potential demand for developing computer-aided diagnosis, especially for these rare diseases. This need can be addressed by exploiting recently advanced artificial intelligence (AI) technologies, particularly deep learning.

However, one significant challenge for developing deep learning models for rare diseases is the difficulty in constructing large, diverse datasets due to their scarcity. Moreover, the rarity of the disease leads to a limited market size for commercially-driven research and development perspectives, leaving diagnostic support systems for rare diseases as *unmet needs* despite their non-negligible clinical significance. Indeed, although several segmentation models for anterior mediastinal tumors have been proposed^5–8^, only a limited number of studies have undergone external validation on data from multiple institutions nationwide^5^, and evidence of clinically applicable and generalizable models is still insufficient.

**Fig. 1:**
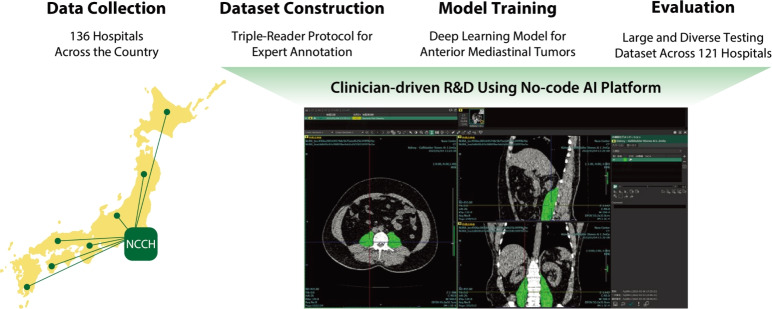
Study Overview. This study comprises four steps. (1) First, we collected a large-scale dataset consisting of 711 CT scans of anterior mediastinal tumors from 136 hospitals across the country. (2) Next, annotation for each tumor was established as reference standards by a triple-reader protocol, including a first expert annotator followed by two expert reviewers. (3) A deep learning-based model was trained using a no-code AI platform to enable clinician-driven research in a standardized and reproducible manner. (4) Finally, the segmentation and detection performance of the trained model was evaluated on an external testing dataset, consisting of large and diverse samples across 121 hospitals.

To overcome these challenges, we developed a deep learning-based diagnostic support system for anterior mediastinal tumors on chest CT images and evaluated its clinical applicability and generalizability (see Fig. 1 for the overview of the study). We collected 711 CT scans of anterior mediastinal tumors from 136 institutions, including the National Cancer Center Hospital (NCCH), a leading cancer-treatment hub in Japan, and 135 hospitals that refer patients to the NCCH. The model was trained on a no-code AI platform^9^, enabling clinician-driven research of diagnostic-support systems in a standardized and reproducible manner. Based on reference standards provided by board-certified diagnostic radiologists, our model achieved clinically applicable segmentation and lesion-wise detection performance for anterior mediastinal tumors, demonstrating broad generalizability across the 121 unique institutions in the external testing dataset.

## Methods

Figure 1 shows the overview of this study, which comprises four steps: collecting a large-scale dataset for the rare disease and evaluating performance from both technical and clinical aspects. Hereinafter, we describe the detailed methodology accordingly.

### Study design

This retrospective observational study collected multi-institutional CT images. Institutional Review Board (IRB) approval was obtained from the National Cancer Center of Japan (approval No. 2023-229). The study adhered to the Declaration of Helsinki and to the Ethical Guidelines for Medical and Health Research Involving Human Subjects in Japan. Because the investigation posed no more than minimal risk and relied on retrospectively collected, de-identified patient data, the requirement for informed consent was waived in accordance with both the ethical guidelines and the IRB approval. Information about the study was publicly disclosed, and patients were offered an opportunity to decline participation through an opt-out procedure. The study was conducted and reported in accordance with the CLAIM statement^10^.

### Data collection

#### Inclusion and exclusion criteria

**Fig. 2:**
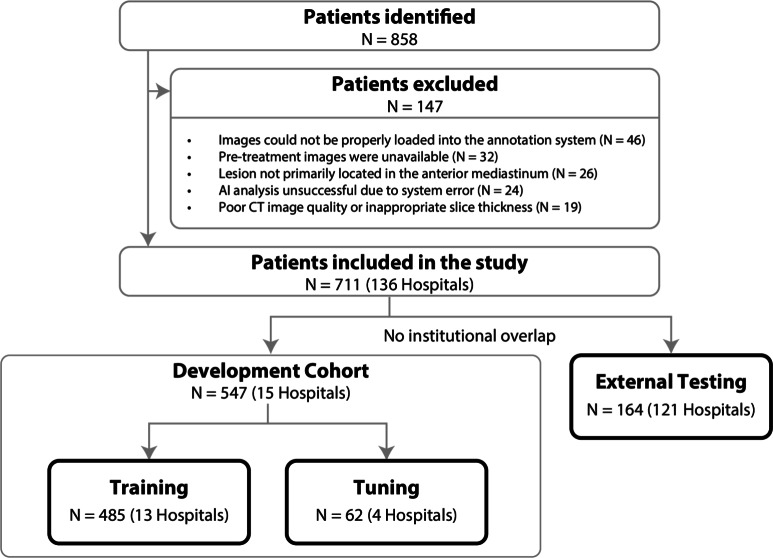
Data Collection and Dataset Splitting. To collect a large-scale dataset for anterior mediastinal tumors, we first identified all diagnosed patients at a leading cancer treatment hub in Japan during a specific period ($N=858$). Then, patients who did not meet quality standards or had any system errors affecting the research were excluded, such as those with poor CT image quality ($N=147$). As a result, a total of 711 cases from 136 hospitals were included in this study. Subsequently, 164 cases from 121 unique hospitals were identified as hold-out cases and assigned to an external testing dataset, with no institutional overlap with the other datasets. Finally, the remaining patients, the development cohort, were randomly split into a training dataset ($N=485$) and a tuning dataset ($N=62$) for model optimization.

Patients diagnosed with anterior mediastinal tumors between April 1, 2000, and March 31, 2024, at NCCH were enrolled (see Fig. 2 for the detailed process of data collection). Inclusion criteria were: age $\ge$ 18 years; availability of complete chest CT images for analysis; and pathologically confirmed diagnosis. Patients with incomplete or poor-quality CT images or missing demographic or clinical data were excluded. Only one chest CT series was analyzed per patient: when the referring institution’s original CT series was available, that series was selected; otherwise, the CT series acquired at NCCH was used. Finally, a total of 711 CT images, each from a different patient, with pathologically diagnosed anterior mediastinal tumors were collected from 136 unique institutes.

#### Image acquisition protocol

At NCCH, CT images were acquired using a single- or multidetector row CT scanner (TCT-900S, Xvigor, Aquilion, Aquilion Prime, Aquilion ONE, and Aquilion Precision; Canon Medical Systems, previously Toshiba Medical Systems, Japan). The acquisition parameters were adjusted based on the scanner models, and the following parameters were consistently applied to all patients: tube voltage, 120 kV; automatic tube current modulation; and slice thicknesses of 5.0, 7.0, or 10.0 mm. An image reconstruction algorithm was applied using a soft-tissue kernel with filtered back projection, hybrid iterative reconstruction, or deep-learning-based reconstruction. The field of view was adjusted to the body size of each patient, which ranged from 320 to 450 mm based on the diameter of the patient.

For other CT images obtained from referral hospitals, the scanner manufacturers included Siemens, Canon Medical Systems, GE Healthcare, Fujifilm (previously Hitachi), and Philips Healthcare. The parameters included detector rows ranging from 4 to 320, tube voltage of 100–135 kV, and slice thickness of 5–10 mm.

### No-code AI platform

For the following processes starting from dataset construction (see Sect. “Dataset construction”) to voxel-wise performance evaluation (see Sect. “Voxel-wise segmentation performance metrics”), we adopted a no-code AI research and development platform, namely SYNAPSE Creative Space^®^ (FUJIFILM Corporation, Tokyo, Japan)^9^, the prototype of which was developed through our collaborative research project. By providing a graphical user-interface to comprehensively support the developmental process of computer-aided diagnosis systems using deep-learning-based algorithms, this software promotes *clinician-driven research and development* in a standardized and reproducible manner.

### Dataset construction

#### Expert annotation for tumor segmentation

Every tumor located in the anterior mediastinum was identified for annotation to define a voxel-wise reference standard for the tumor segmentation. The definition of the anterior mediastinum was based on the ITMIG classification^11^, which typically includes the area in front of the heart and the trachea. Note that extending tumors originating from the anterior mediastinum to adjacent regions, such as the middle mediastinum, were included; however, those exclusively in the middle or posterior mediastinum were excluded.

Expert annotation for tumor segmentation was performed according to the following *triple-reader protocol*. First, a board-certified thoracic surgeon or a board-certified radiology technologist manually annotated the anterior mediastinal tumor regions on each CT image. Then, the initial annotations were reviewed by two board-certified diagnostic radiologists, each with more than 20 years of experience in thoracic imaging. In cases of disagreement during the review process, consensus was reached through discussion. Finally, all annotations for tumor segmentation were confirmed to meet clinical standards. This rigorous process ensured that each tumor annotation was thoroughly validated by three expert readers (i.e., first expert annotator followed by two expert reviewers), enhancing the reliability of our reference standards.

#### Dataset splitting

In our study, one CT image was obtained per patient, thus the terms ‘patient case’ and ‘CT image’ can be used interchangeably (see Fig. 2 for the dataset splitting process). For the annotated 711 CT images from 136 institutes, we first separated 164 CT images scanned at 121 referral institutions as hold-out cases. Then, the remaining CT images, as a development cohort, were randomly split into a *training dataset* ($N=485$ cases from 13 institutions) and a *tuning dataset* ($N=62$ cases from 4 institutions). The hold-out cases were assigned as *external testing dataset* ($N=164$ cases from 121 institutions). Importantly, there is no overlap at the institutional level between the external testing dataset and the other two datasets, the training and tuning datasets, which ensures the model could be generalizable to populations beyond a single institute. Additionally, all CT images in the external testing dataset had a slice thickness of 5 mm; this uniformity was not imposed as an inclusion criterion but naturally reflected the predominance of such imaging protocols in real-world clinical practice. Note that the dataset was also partitioned at the patient level, ensuring that images from the same patient were not included in multiple datasets.

#### Data pre-processing

Before model training and evaluation, pixel values of CT images were rescaled to the [0, 1] range using the minimum and maximum signal values in the dataset.

**Fig. 3:**
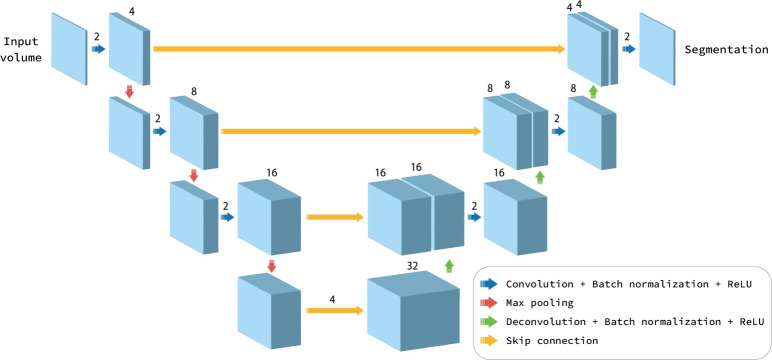
Segmentation Model Architecture. A three-dimensional U-Net-based deep neural network was trained and evaluated in this study. The network consists of four hierarchical levels in both encoding and decoding paths, with skip connections between corresponding levels. Each level contains multiple processing blocks applied sequentially. In the encoding path, each block consists of convolution, batch normalization, and ReLU activation, with max pooling operations between levels that reduce spatial resolution. In the decoding path, each block consists of deconvolution, batch normalization, and ReLU activation, with upsampling operations that increase resolution. Skip connections transfer feature maps from encoding to decoding paths to preserve spatial information. Finally, segmentation masks with the same resolution as the input image for the anterior mediastinal tumor are predicted as the output.

### Model training

#### Deep neural network architecture

We adopted a three-dimensional (3D) U-Net architecture^12,13^, which is widely used for medical image analysis. The network consists of four hierarchical levels in both encoding and decoding paths, with skip connections between corresponding levels. Each encoding block contains a 3D convolution layer followed by batch normalization, ReLU activation, and max pooling, whereas each decoding block contains a 3D deconvolution layer followed by batch normalization and ReLU activation. See Fig. 3 for the detailed network architecture.

Note that the output of the neural network is a binary mask indicating tumor regions in the input image (0 for normal tissue, 1 for the tumor). While segmentation performance is evaluated directly using this prediction mask, the evaluation of lesion detection performance requires additional postprocessing as described in Sect. “Post-processing for separating lesion-wise components”, where we evaluate the overlap between 3D components decomposed into a per-lesion basis.

#### Training settings

The model parameters were initialized with a uniform Glorot distribution for the convolutional and deconvolutional weights, and the biases were initialized to zero. In the training step, data augmentation techniques were applied as follows: (1) random rotations between –10° to +10° on the axial plane; (2) scaling along the axial axes independently by a factor of 0.8 to 1.2; and (3) random cropping. Random cropping was applied during training to ensure that each batch contained both the foreground and background. The center of the crop for the first data point was chosen uniformly from the entire image. The batch size was set to 3. The model was trained using the Adam optimizer with a constant learning rate of 0.001, $\beta _1$ of 0.9, and $\beta _2$ of 0.999. Early stopping was not employed. For the objective function, we employed the Dice loss.

During the training, Dice score, which reflects the overlap between the model prediction and reference standard (see Eq. 1 for the definition), was used as a metric to monitor progress. Finally, the model that achieved the highest Dice score on the tuning dataset was selected for the evaluation using the external testing dataset.

### Evaluation

To evaluate the trained model’s performance from both technical and clinical aspects, we employed two types of metrics: *voxel-wise performance metrics* and *lesion-wise performance metrics*. These metrics were calculated on the external testing dataset. Note that since the segmentation labels as reference standards were annotated by the triple-reader protocol, (see Sect. “Expert annotation for tumor segmentation”), these metrics reflect the agreement between the model prediction and expert interpretation on CT images for the anterior mediastinal tumors.

#### Voxel-wise segmentation performance metrics

Voxel-wise performance metrics primarily focus on the voxel-wise overlap between model predictions and reference standards, which measures how accurately the model predicts whether each voxel contains tumor or not. These metrics were calculated for each CT image series as a whole, providing a comprehensive evaluation across the entire volumetric dataset. We evaluated our model using four standard metrics: Dice score, Intersection over Union (IoU), Precision, and Recall.

The *Dice score* (also known as *F1-score*) measures the overall overlap between prediction and reference standards, and is defined as:

1 $$\begin{aligned} \text {Dice} = \frac{2|X \cap Y|}{|X| + |Y|} = \frac{2 \times \text {TP}}{2 \times \text {TP} + \text {FP} + \text {FN}}, \end{aligned}$$

where *X* represents the predicted segmentation and *Y* represents the segmentation labels as reference standards. True positives (TP) refer to the voxels that are correctly predicted as tumor, false positives (FP) are voxels incorrectly predicted as tumor, and false negatives (FN) represent voxels that are labeled as tumor in the reference standards but are missed by the model.

*IoU*, also known as the *Jaccard index*, quantifies the ratio of the overlap between the predicted segmentation and the reference standard to their union, and is defined as:

2 $$\begin{aligned} \text {IoU} = \frac{|X \cap Y|}{|X \cup Y|} = \frac{\text {TP}}{\text {TP} + \text {FP} + \text {FN}}. \end{aligned}$$

While the Dice score and IoU are closely related, IoU tends to be more stringent in penalizing mismatches, making it a useful complementary metric for segmentation performance evaluation.

*Precision* quantifies the proportion of correctly identified tumor voxels among all voxels predicted as tumor, and is calculated as:

3 $$\begin{aligned} \text {Precision} = \frac{\text {TP}}{\text {TP} + \text {FP}}. \end{aligned}$$

*Recall* (also known as sensitivity) measures the proportion of actual tumor voxels that were correctly identified by the model, and is defined as:

4 $$\begin{aligned} \text {Recall} = \frac{\text {TP}}{\text {TP} + \text {FN}}. \end{aligned}$$

#### Post-processing for separating lesion-wise components

To evaluate the model’s lesion-wise detection performance, we further processed the predicted segmentation masks to separate lesion-wise components as follows. First, we extracted 3D connected-components from the prediction masks. Then, we removed small connected regions containing fewer than 10 voxels to reduce noise in the segmentation results. Finally, we applied the same 3D connected-component separation to the reference standards, enabling lesion-wise overlap analysis.

#### Lesion-wise detection performance metrics

Beyond the accuracy at the image series level, the capability to detect individual lesions is crucial for a diagnostic support system to help clinicians avoid overlooking disease lesions. Thus, we conducted performance evaluations at the individual lesion level by measuring the overlap between the model prediction and reference standards. This approach treats each lesion as an independent evaluation unit rather than the entire image series, which was the focus of the above-mentioned voxel-wise performance metrics (see Sect. “Voxel-wise segmentation performance metrics”).

The *Free-response Receiver Operating Characteristic* (FROC) curve based on IoU thresholds was conducted to provide a comprehensive assessment of the model’s lesion-detection capability. In detail, we first identified 3D connected-components of segmentation masks both from model prediction and reference standard (see Sect. “Post-processing for separating lesion-wise components”). Then, we applied a systematic IoU threshold sweep from 0.05 to 0.95 with a step size of 0.05. For each threshold, we calculated the sensitivity and the number of false positives per scan (FP/scan), using one-to-one matching between predicted and reference standard lesions. In this evaluation, a predicted lesion was considered a true positive if its IoU with a reference standard lesion exceeded the given threshold. All unmatched predictions were treated as false positives, and all unmatched reference standard lesions were treated as false negatives. By plotting sensitivity against FP/scan across varying IoU thresholds, the resulting FROC curve illustrates the trade-off between detection sensitivity and the number of false alarms.

In the context of FP/scan in the FROC curve, the calculation of ‘false positives’ is based on the lesion level, meaning that any 3D-connected component in the model prediction that does not overlap with any reference standard component is considered a false positive. Note that its calculation differs from those in the context of voxel-wise segmentation metrics in Sect. “Voxel-wise segmentation performance metrics”.

## Results

**Table 1:** Patient demographics and pathological diagnosis across datasets.

Variable	Training ($N=485$)	Tuning ($N=62$)	External testing ($N=164$)
Age (Median, range)	58.0 (20–87)	60.5 (23–87)	58.0 (21–85)
Pathological diagnosis (Number of cases, %)			
Thymoma	291 (60.0%)	39 (62.9%)	74 (45.1%)
Thymic carcinoma	103 (21.2%)	13 (21.0%)	71 (43.3%)
Lymphoma	59 (12.2%)	6 (9.7%)	6 (3.7%)
Thymic cyst	30 (6.2%)	4 (6.4%)	10 (6.1%)
Teratoma	2 (0.4%)	0 (0.0%)	1 (0.6%)
Carcinoid	0 (0.0%)	0 (0.0%)	1 (0.6%)
Sarcomatous transformation of teratoma	0 (0.0%)	0 (0.0%)	1 (0.6%)

**Table 2:** CT scanner manufacturers in the external testing dataset.

CT scanner manufacturer	External testing ($N=164$)
Canon medical systems	80 (48.8%)
GE healthcare	37 (22.5%)
Siemens	35 (21.3%)
Fujifilm	6 (3.7%)
Philips healthcare	6 (3.7%)

### Patient characteristics

As indicated in Fig. 2, a defining strength of this study design lies in the rigorous separation and institutional diversity of the datasets. The development cohort consisted of a training dataset ($N=485$ from 13 institutions) and a tuning dataset ($N=62$ from 4 institutions). In sharp contrast, the external testing dataset comprised 164 cases collected from 121 distinct institutions, with absolutely no institutional overlap between the development and testing cohorts. This extensive multi-institutional composition of the external test set provides a robust foundation for evaluating the model’s generalizability across different clinical environments.

The demographic and clinical characteristics of patients across the training, tuning, and external testing datasets are summarized in Table 1. Regarding the pathological distribution, the major histological types of anterior mediastinal tumors were thymoma ($N=404$, 56.8%), thymic carcinoma ($N=187$, 26.3%), malignant lymphoma ($N=71$, 9.9%), thymic cyst ($N=44$, 6.2%), and carcinoid tumor ($N=1$, 0.1%). These tumors represent rare entities that pose substantial diagnostic challenges.

To further assess the model’s robustness against acquisition variability, Table 2 highlights the imaging characteristics of the external testing dataset. Reflecting the wide range of contributing facilities (121 institutions), the dataset encompasses an extensive diversity of CT scanner manufacturers. While detailed acquisition parameters for the training and tuning datasets are described in Sect. “Image acquisition protocol”, they are omitted from Table 2 to maintain the focus on the external testing of model performance. Note that for other acquisition parameters not detailed in the table, the external testing dataset consistently utilized a slice thickness of 5 mm and soft-tissue reconstruction kernels, adhering to standard thoracic imaging protocols.

### Segmentation performance on voxel-wise metrics

**Table 3:** Detailed segmentation performance metrics by subgroup. Values are presented as mean ± standard deviation (95% confidence interval).

Subgroup	No. of cases	Dice score	IoU	Precision	Recall
Overall	164	0.82±0.15 (0.79–0.84)	0.72±0.17 (0.69–0.74)	0.85±0.12 (0.83–0.87)	0.82±0.19 (0.79–0.84)
*Scanner manufacturer*					
Canon Medical Systems	80	0.89±0.04 (0.86–0.93)	0.82±0.07 (0.76–0.87)	0.91±0.01 (0.90–0.91)	0.89±0.08 (0.83–0.94)
GE Healthcare	37	0.81±0.20 (0.73–0.87)	0.71±0.21 (0.64–0.78)	0.83±0.17 (0.76–0.88)	0.82±0.22 (0.74–0.89)
Siemens	35	0.82±0.13 (0.77–0.86)	0.72±0.16 (0.66–0.77)	0.86±0.07 (0.84–0.89)	0.82±0.18 (0.75–0.87)
Fujifilm	6	0.88±0.04 (0.85–0.91)	0.80±0.06 (0.74–0.85)	0.89±0.02 (0.86–0.90)	0.88±0.06 (0.83–0.94)
Philips Healthcare	6	0.85±0.08 (0.77–0.92)	0.75±0.13 (0.64–0.85)	0.92±0.03 (0.89–0.94)	0.80±0.14 (0.68–0.91)
*Tumor size*					
Large ($> 8$ cm)	68	0.86±0.09 (0.84–0.88)	0.77±0.12 (0.74–0.80)	0.86±0.08 (0.84–0.88)	0.87±0.12 (0.84–0.90)
Medium ($5\text {-}\text {-}8$ cm)	57	0.80±0.19 (0.75–0.85)	0.71±0.20 (0.65–0.76)	0.86±0.12 (0.83–0.89)	0.79±0.23 (0.73–0.85)
Small ($< 5$ cm)	39	0.77±0.16 (0.71–0.81)	0.65±0.17 (0.59–0.70)	0.81±0.17 (0.75–0.86)	0.75±0.19 (0.69–0.81)
*Histology*					
Thymoma	74	0.82±0.16 (0.78–0.86)	0.73±0.18 (0.68–0.77)	0.86±0.13 (0.82–0.89)	0.81±0.19 (0.77–0.86)
Thymic carcinoma	71	0.82±0.15 (0.78–0.85)	0.72±0.16 (0.68–0.76)	0.84±0.12 (0.81–0.87)	0.83±0.18 (0.79–0.87)
Thymic cyst	10	0.77±0.07 (0.72–0.82)	0.63±0.10 (0.57–0.70)	0.85±0.07 (0.80–0.89)	0.72±0.11 (0.64–0.78)
Lymphoma	6	0.78±0.22 (0.58–0.90)	0.68±0.23 (0.47–0.82)	0.83±0.05 (0.79–0.87)	0.80±0.28 (0.54–0.95)
Others	3	0.88±0.04 (0.84–0.94)	0.78±0.07 (0.72–0.90)	0.90±0.02 (0.87–0.93)	0.86±0.07 (0.78–0.96)

We measured the voxel-wise performance metrics (see Sect. “Voxel-wise segmentation performance metrics”), which focus on the voxel-wise overlap between model prediction and reference standard at the CT-image level. On the external testing dataset, the model achieved average ± standard deviation of Dice score, IoU, Precision, and Recall values of $0.82 \pm 0.15$, $0.72 \pm 0.17$, $0.85 \pm 0.12$, and $0.82 \pm 0.19$, respectively. To evaluate the model’s robustness considering the diversity of the external testing dataset, we further performed stratified analyses based on CT scanner manufacturer, tumor size (defined as the maximum diameter measured in the axial plane), and histology (see Table 3). Notably, the model maintained a mean Dice score of 0.77 or higher across all subgroup analyses, suggesting that it possesses high generalizability across large and diverse samples collected from 121 hospitals throughout the country.

### Detection performance on lesion-wise metrics

**Table 4:** Detailed detection performance metrics by subgroup. Values are presented as mean ± standard deviation (95% confidence interval).

Subgroup	No. of cases	IoU = 0.1		IoU = 0.5	
		Sensitivity	FP per scan	Sensitivity	FP per scan
Overall	164	0.95 ± 0.01 (0.92–0.98)	0.51 ± 0.08 (0.37–0.68)	0.87 ± 0.02 (0.81–0.92)	0.61 ± 0.08 (0.46–0.78)
*Scanner manufacturer*					
Canon medical systems	80	0.96 ± 0.02 (0.90–1.00)	0.60 ± 0.13 (0.35–0.88)	0.84 ± 0.04 (0.75–0.92)	0.72 ± 0.14 (0.46–1.02)
GE healthcare	37	0.92 ± 0.05 (0.80–1.00)	0.43 ± 0.08 (0.27–0.62)	0.87 ± 0.06 (0.73–0.97)	0.48 ± 0.09 (0.32–0.67)
Siemens	35	0.97 ± 0.02 (0.91–1.00)	0.50 ± 0.19 (0.17–0.94)	0.91 ± 0.04 (0.81–1.00)	0.55 ± 0.19 (0.23–0.97)
Fujifilm	6	1.00 ± 0.00 (1.00–1.00)	0.16 ± 0.15 (0.00–0.50)	1.00 ± 0.00 (1.00–1.00)	0.16 ± 0.15 (0.00–0.50)
Philips healthcare	6	1.00 ± 0.00 (1.00–1.00)	0.33 ± 0.19 (0.00–0.66)	0.85 ± 0.10 (0.66–1.00)	0.50 ± 0.20 (0.16–0.83)
*Tumor size*					
Large ($> 8$ cm)	68	0.95 ± 0.02 (0.89–1.00)	0.65 ± 0.17 (0.34–1.04)	0.90 ± 0.04 (0.80–0.98)	0.71 ± 0.18 (0.40–1.11)
Medium ($5\text {-}\text {-}8$ cm)	57	0.96 ± 0.02 (0.91–1.00)	0.56 ± 0.10 (0.36–0.77)	0.84 ± 0.04 (0.75–0.93)	0.68 ± 0.11 (0.47–0.91)
Small ($< 5$ cm)	39	0.94 ± 0.04 (0.85–1.00)	0.21 ± 0.06 (0.07–0.34)	0.84 ± 0.06 (0.70–0.97)	0.31 ± 0.07 (0.15–0.47)
*Histology*					
Thymoma	74	0.97 ± 0.02 (0.92–1.00)	0.52 ± 0.12 (0.31–0.78)	0.86 ± 0.04 (0.77–0.94)	0.63 ± 0.13 (0.39–0.91)
Thymic carcinoma	71	0.93 ± 0.03 (0.86–0.98)	0.52 ± 0.13 (0.29–0.80)	0.88 ± 0.04 (0.78–0.95)	0.57 ± 0.13 (0.35–0.84)
Thymic cyst	10	1.00 ± 0.00 (1.00–1.00)	0.60 ± 0.20 (0.20–1.00)	0.81 ± 0.10 (0.61–1.00)	0.80 ± 0.23 (0.30–1.20)
Lymphoma	6	1.00 ± 0.00 (1.00–1.00)	0.50 ± 0.20 (0.16–0.83)	0.83 ± 0.15 (0.50–1.00)	0.66 ± 0.30 (0.16–1.33)
Others	3	1.00 ± 0.00 (1.00–1.00)	0.00 ± 0.00 (0.00–0.00)	1.00 ± 0.00 (1.00–1.00)	0.00 ± 0.00 (0.00–0.00)

**Fig. 4:**
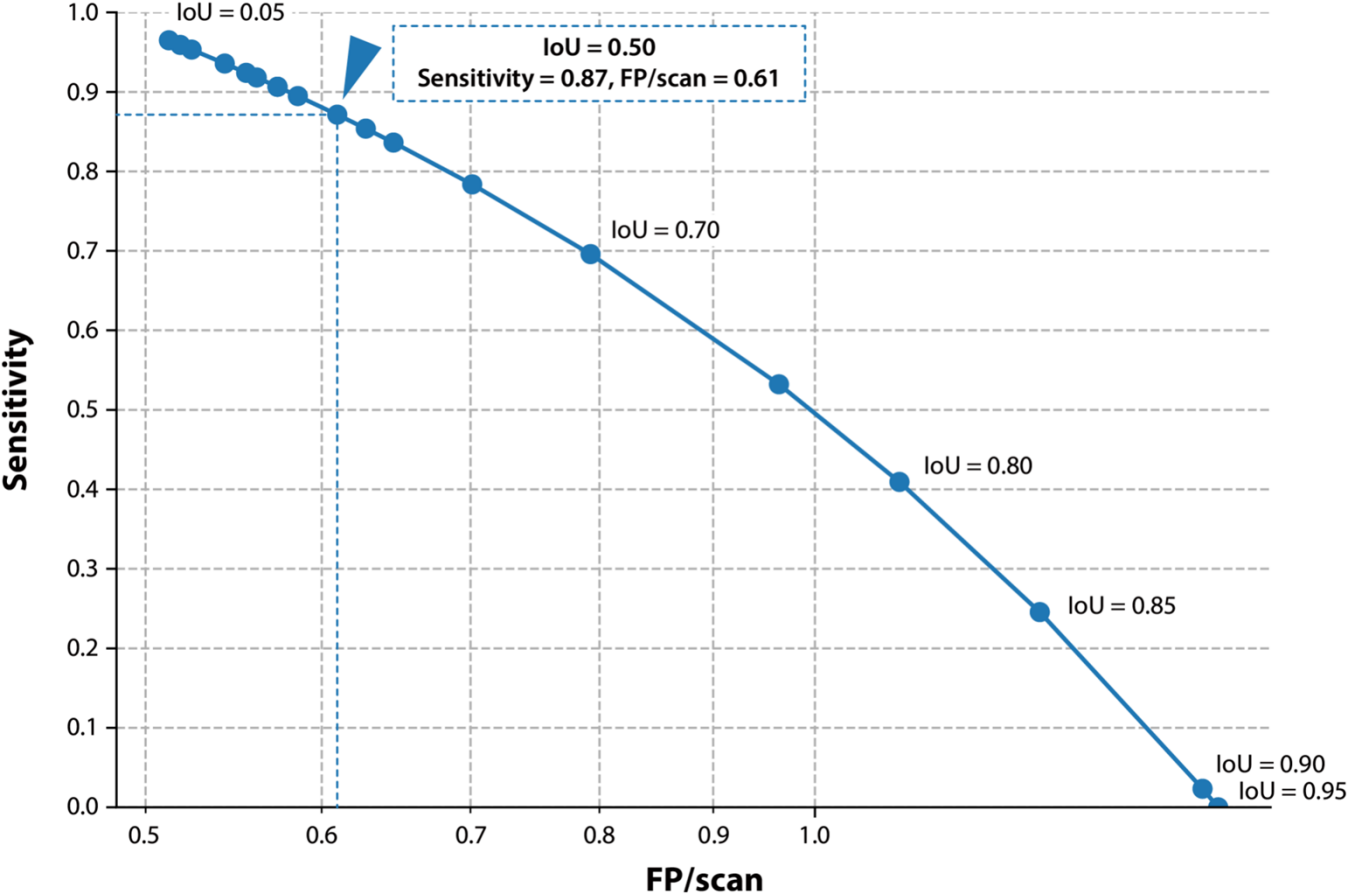
Free-response receiver operating characteristic for the lesion detection performance. To evaluate the lesion detection performance of the trained model, we identified three-dimensional connected-components that are separated from segmentation masks of both reference standard and model prediction. Then, by sweeping Intersection over Union (IoU) threshold, we calculated the sensitivity and the number of false positives per scan (FP/scan), using one-to-one matching between components. Our model achieved high-sensitivity and low-FP characteristics, which will be advantageous for cancer screening purposes, and its high sensitivity still remains even under a strict IoU threshold of 0.50, as shown by the arrowhead.

Based on the 3D connected-components of the segmentation masks, we also evaluated lesion-wise performance metrics to assess how accurately the model can detect each anterior mediastinal tumor on CT images. Figure 4 shows the FROC curve, in which the IoU thresholds were systematically swept from 0.05 to 0.95 with a step size of 0.05 (see Sect. “Lesion-wise detection performance metrics” for detailed evaluation methods). Notably, our model achieved high sensitivity ranging from 0.95 to 0.97 with a low number of FP/scan around 0.51–0.52 when the IoU threshold was between 0.05 and 0.15. This high-sensitivity and low-FP characteristic is particularly advantageous for cancer screening purposes. Furthermore, even under a stricter IoU threshold of 0.50, the model maintained high sensitivity of 0.87 ± 0.02 (mean ± standard deviation) [95% Confidence Interval (CI): 0.81–0.92] with relatively low FP/scan of 0.61 ± 0.08 (95% CI: 0.45–0.77) (see the arrowhead in Fig. 4). To further investigate the robustness of the detection performance across different acquisition and clinical conditions, we conducted subgroup analyses stratified by CT scanner manufacturer, tumor size (defined as the maximum diameter measured in the axial plane), and histology at IoU thresholds of 0.1 and 0.5, respectively (see Table 4). These results collectively indicate that the model is capable of robust lesion detection even under varying conditions and strict matching criteria, making it suitable for both screening and diagnostic support applications.

### Per-case analysis of successes and failures

**Fig. 5:**
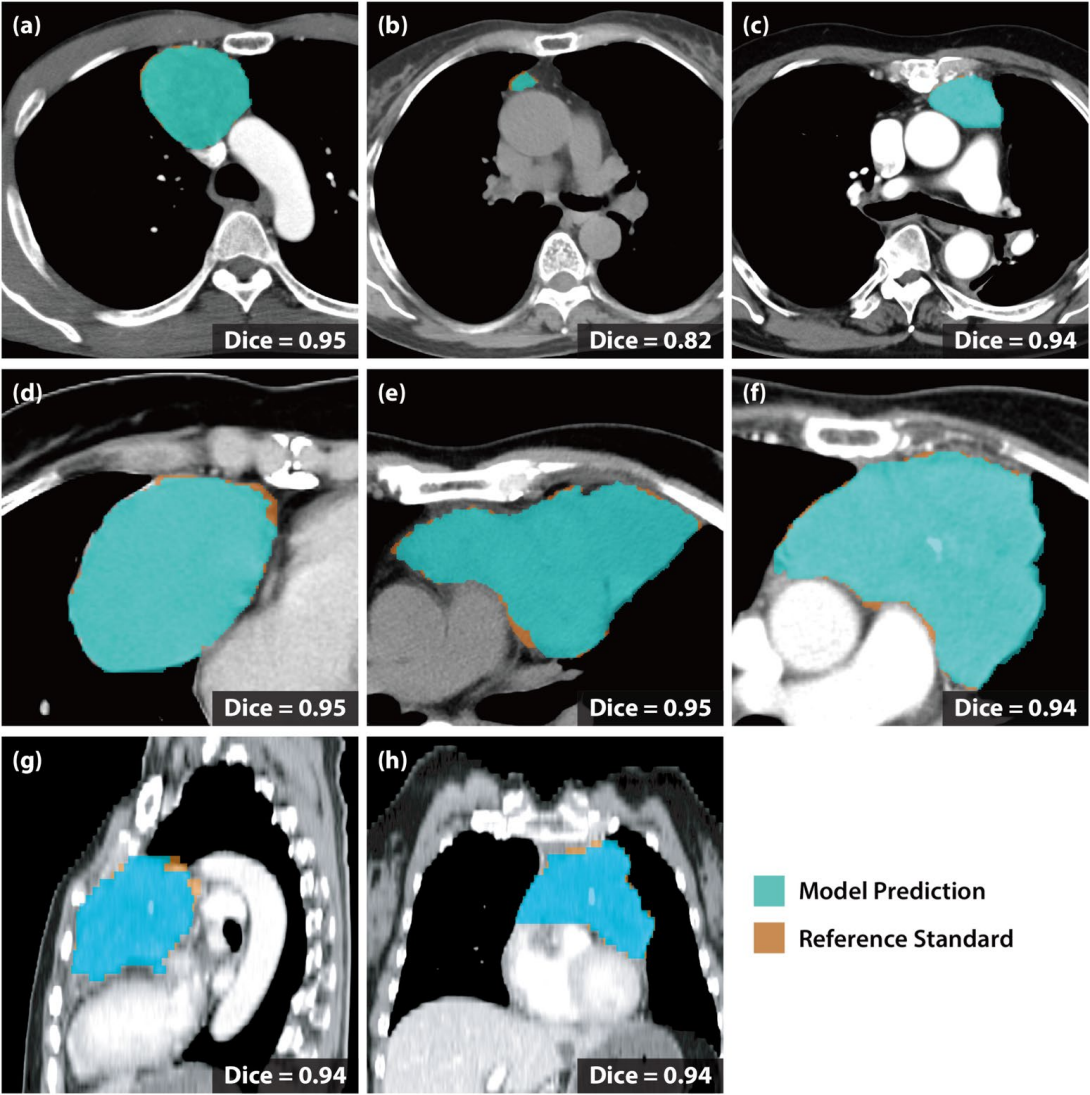
Per-case qualitative analysis of successful cases. Six representative CT images with Dice scores above 0.70 show that the model prediction (shown by the blue regions) closely matched the manual annotations (shown by the orange regions), indicating accurate tumor delineation (**a–f**). (**g**) and (**h**) display the sagittal and coronal views of (**f**), respectively.

**Fig. 6:**
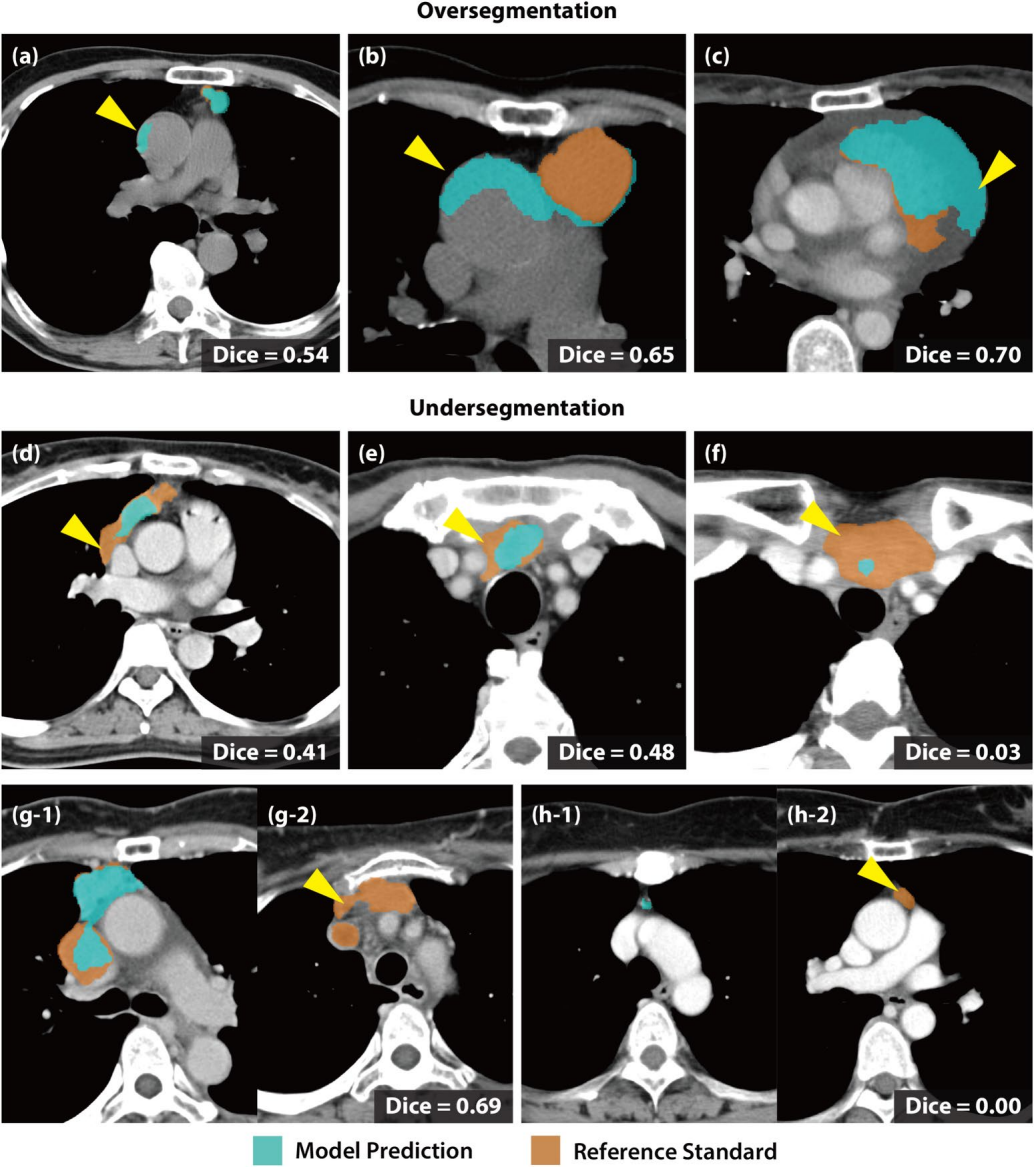
Per-Case Qualitative Analysis of Failure Cases. We divided the error categories into oversegmentation (**a–c**) and undersegmentation (**d–h**). The examples of oversegmentation show the false detection of normal tissues as part of the tumor region, such as ascending aorta (**a–b**) or pericardial effusion (**c**), shown by the arrowheads. Undersegmented regions (shown by the arrowheads) include the insufficient model prediction that includes only a portion of the actual lesion (**d–g**) or missing the target entirely (**h**). Subfigures (**g1–g2**) and (**h1–h2**) show different slices from the same patients, respectively.

Finally, we qualitatively assessed the successful and failure cases. As shown in Fig. 5a–f, the model precisely identified the tumor boundaries whose locations are rather confined within the anterior mediastinal region. In contrast, we observed two failure modes of the model prediction: *oversegmentation* and *undersegmentation*. The model oversegmented normal tissues, such as the ascending aorta or pericardial effusion, as part of the tumor region (see Fig. 6a–c). Undersegmentation involved partial detection of the tumor or complete misses in a limited number of cases, particularly in low-contrast cases or small tumors (see Fig. 6d–h). These findings suggest that radiologists should prioritize manual review for small or low-contrast tumors, while leveraging the model’s high sensitivity (0.87 overall) for initial screening of larger or high-contrast tumors.

### Quantitative analysis of failure modes

To quantitatively substantiate our qualitative observations regarding failure modes, we conducted a radiomics-based analysis^14^ comparing the cases with the lowest segmentation performance (bottom 10% of cases based on Dice score, $N=17$) against the cases with the highest performance (top 10% of cases, $N=17$) in the external testing dataset. We extracted shape and first-order intensity features from the reference standard masks to characterize the tumor properties.

The analysis revealed statistically significant differences in tumor size between the two groups. Specifically, the high-performance group exhibited significantly larger values in Voxel Volume ($p = 0.003$*) and Maximum 3D Diameter ($p = 0.025$*) compared to the low-performance group (* indicates $p < 0.05$). Regarding intensity features, while Total Energy was significantly higher in the high-performance group ($p = 0.009$*)—reflecting the larger tumor volume involved in the calculation—basic intensity metrics such as Mean ($p = 0.42$) and Median ($p = 0.33$) CT attenuation did not show significant differences. These quantitative results support our qualitative finding that model performance tends to degrade specifically in smaller tumors, whereas the model maintains robust performance for larger lesions regardless of their average density.

## Discussion

Despite the potential need for computer-aided diagnosis in rare diseases, their low prevalence makes it difficult to construct the large, diverse datasets required for training and evaluating deep-learning models. Anterior mediastinal tumors represent such rare entities and pose significant diagnostic challenges. In this study, by gathering referral cases at a leading cancer-treatment center in Japan, we collected 711 cases from 136 institutions with diverse characteristics (see Sect. “Patient characteristics”). Moreover, a clinician-driven workflow—using a no-code AI platform for model training and evaluation—ensured that our research process was both standardized and reproducible. As a result, our model achieved clinically applicable performance for both segmentation and detection on the external testing dataset. Crucially, this dataset comprised 164 CT scans from 121 previously unseen institutions. The model’s consistently strong performance across such a large number of independent facilities provides compelling evidence of its robust generalizability and broad applicability. This approach effectively addressed the data scarcity challenge inherent to rare diseases, enabling robust model development for anterior mediastinal tumors.

These technical achievements can be translated into clinical practice to enhance diagnostic efficiency for anterior mediastinal tumors. For instance, the high segmentation performance, with a Dice score of 0.82, enables precise tumor delineation despite their confusing appearance and spatial adjacency to normal tissue (see Sect. “Segmentation performance on voxel-wise metrics”). This precision supports critical clinical tasks, including tumor volume measurement for disease staging, radiotherapy planning for accurate target definition, and preoperative planning for surgical resection. Regarding practical deployment, the model’s output (i.e., segmentation masks) can be converted into DICOM RT Structure Sets or Secondary Capture objects for seamless handoff to Picture Archiving and Communication Systems (PACS) or treatment planning software. Moreover, the model’s detection capability, with a high sensitivity of 0.87 and a low false-positive rate of 0.61 per scan (see Sect. “Detection performance on lesion-wise metrics”), makes it well-suited as a first- or second-reader tool. In this workflow, a “human-in-the-loop” approach is essential: radiologists perform a rapid false-positive triage to dismiss non-tumor candidates before confirming the final report. Additionally, the deployment interface can support an adjustable thresholding policy, allowing users to calibrate the probability threshold to prioritize sensitivity for screening or precision for volumetric analysis, depending on the clinical intent.

A key strength of our work is the exclusive focus on anterior mediastinal tumors, validated on a large, heterogeneous test set spanning 121 hospitals. In this domain, Tang et al. developed CAIMEN—a federated-learning–based AI system trained on 7825 mediastinal neoplasm cases from 24 Chinese centers—demonstrating high performance on 1216 scans from five institutions^5^. While their study offers valuable insights into federated learning for a broad range of mediastinal neoplasms, direct numerical comparisons should be approached with caution due to inherent differences in target diseases and dataset distributions. Distinct from their comprehensive scope covering all mediastinal compartments, our study specifically targets anterior mediastinal tumors and employs a rigorous triple-reader protocol to establish a clinically meaningful voxel-level reference standard (see Sect. “Expert annotation for tumor segmentation”). Furthermore, our external testing dataset is characterized by extensive institutional diversity across scanner vendors and imaging protocols from 121 different institutions (see Table 2), which contributes to a robust evaluation of the model’s applicability and generalizability in varied clinical settings.

Adopting clinician-driven research and development using a no-code AI platform for a computer-aided diagnosis system is also an important feature of this study (see Fig. 1). Even though there is increasing attention in the application of deep learning technologies in medicine, an interdisciplinary approach between data scientists and clinicians is often difficult due to many reasons such as the shortage of personnel and confidentiality of medical data. For example, Tang et al. employed a federated learning framework, which is technically complicated and not accessible for most biomedical researchers^5^. The use of a no-code AI platform can help clinicians participate in the entire process from data-preparation to the model development and evaluation^15–17^, by promoting the development of computer-aided diagnosis system in an environment closer to the actual clinical practice.

Furthermore, clinician-driven research and development can be better aligned with the recent shift toward *data-centric AI*^18^, which emphasizes the importance of data quality over model complexity. The use of the no-code AI platform allowed us to allocate more resources to data preparation rather than sophisticated model development, yet still achieve high-performance results. Indeed, the resultant model performance with a Dice score of 0.82 is high enough to be comparable to other research that focused on the technical aspects of mediastinal tumor segmentation. These comparative studies include a two-staged 3D ResUNet network combined with lung segmentation^6^, nnU-Net with automatic optimization^7^, or CLIP (Contrastive Language-Image Pre-training)-prompt guided cross-attention segmentation network^8^.

Future research should incorporate qualitative factors such as histopathological subtypes and treatment-planning support building on top of the segmentation results of the proposed model. In this line of research, several studies have explored classification of mediastinal lesions by integrating multimodal information, such as imaging features and clinical information^19–21^. For example, recent work using a hybrid transformer model that integrates low-dose CT images with clinical data demonstrated improved surgical eligibility predictions for mediastinal tumors^22^. Such multimodal approaches are advancing more to the integration of CT images with circulating tumor cells for the classification of mediastinal lesions^23^.

There are several limitations in this study. First, despite the heterogeneity of the datasets, our data collection protocol was limited to referral cases to a single institute (i.e., NCCH), raising potential concerns about selection bias for these referral cases. Second, despite the large dataset for rare entities, certain very rare histological entities such as carcinoid and teratoma with sarcomatous transformation are limited in number (see Table 1). Hence, the performance of the model for these subtypes could not be reliably assessed. Further research will include prospective, multi-institutional cohorts with comparisons to radiologists’ diagnostic capabilities to establish the actual clinical usability of the model.

## Conclusion

Our model achieved clinically applicable segmentation and detection performance for anterior mediastinal tumors, demonstrating generalizability across 121 institutions and overcoming data-scarcity challenges. Its high sensitivity (0.87) and precise delineation (Dice 0.82) support its use as a first-reader tool for screening and a second-reader tool for tumor contouring, aiding tumor volume measurement, radiotherapy planning, and preoperative planning. Detailed error analysis revealed lower performance for small and low-contrast tumors, requiring manual review to ensure accuracy. Prospective studies are needed for clinical validation.

## Data Availability

The datasets generated and/or analyzed during the current study are not publicly available due to patient privacy concerns but are available from the corresponding author on reasonable request. The trained model weights are available at https://huggingface.co/hirwatan/FFSCS.
